# Functional Phytochemicals Cooperatively Suppress Inflammation in RAW264.7 Cells

**DOI:** 10.3390/nu18030376

**Published:** 2026-01-23

**Authors:** Kaori Terashita, Masato Kohakura, Katsura Sugawara, Shinichi Miyagawa, Gen-ichiro Arimura

**Affiliations:** Department of Biological Science and Technology, Faculty of Advanced Engineering, Tokyo University of Science, Tokyo 125-8585, Japan

**Keywords:** anti-inflammatory, capsaicin, 1,8-cineole, β-eudesmol, menthol, transient receptor potential (TRP)

## Abstract

Background: Chronic inflammation contributes to the development of lifestyle-related diseases, and dietary phytochemicals are recognized as important modulators of inflammatory responses. However, the synergistic anti-inflammatory effects of phytochemical combinations and their underlying mechanisms remain insufficiently understood. Methods: The anti-inflammatory activities of menthol (ME), 1,8-cineole (CI), β-eudesmol (EU), and capsaicin (CA) were evaluated in lipopolysaccharide (LPS)-stimulated RAW264.7 macrophages. Pro-inflammatory gene expression was quantified by quantitative PCR, intracellular Ca^2+^ signaling was assessed by calcium imaging, and the involvement of transient receptor potential (TRP) channels was examined using selective inhibitors. Synergistic effects were analyzed based on changes in half-maximal effective concentrations (EC_50_). Results: All compounds suppressed LPS-induced pro-inflammatory genes, including tumor necrosis factor-alpha (*Tnf*) and interleukin-6 (*Il6*), in a dose-dependent manner, with CA showing the lowest EC_50_ for *Tnf* expression (0.087 µM). Notably, combinations of CA with ME or CI exhibited strong synergy, reducing their EC_50_ values by 699-fold and 154-fold, respectively, without cytotoxicity. These effects likely resulted from the synergic interaction between ME/CI-induced TRP-mediated signaling and CA-activated, TRP-independent signaling. Conclusions: Specific combinations of plant-derived functional components can markedly enhance anti-inflammatory efficacy, supporting dietary strategies that harness multiple phytochemicals for inflammation control and disease prevention.

## 1. Introduction

Inflammation is a fundamental physiological response that protects the body against infections and tissue injuries. When dysregulated or persistent, however, inflammation contributes to the development of numerous diseases, including atherosclerosis, type 2 diabetes, rheumatoid arthritis, and cancer [1]. Among the immune cells involved, macrophages play a central role by releasing pro-inflammatory cytokines and mediators—such as nitric oxide, tumor necrosis factor-alpha (TNF-α), and interleukin-6 (IL-6)—particularly upon stimulation by lipopolysaccharide (LPS) [2]. Because these macrophage-derived mediators drive both acute and chronic inflammation, identifying factors that regulate macrophage activation is essential for understanding inflammatory disease mechanisms and developing new anti-inflammatory strategies [3].

To investigate these processes experimentally, the murine macrophage-like cell line RAW264.7 is commonly employed as an in vitro model of inflammation. It provides a reproducible and biologically relevant system for evaluating the effects of bioactive compounds on macrophage-mediated responses [4]. Increasing attention has been directed toward plant-derived functional compounds—such as flavonoids (e.g., quercetin, kaempferol), polyphenols (e.g., resveratrol, curcumin), and terpenoids—that exhibit anti-inflammatory activities by modulating key signaling pathways, including nuclear factor kappa B (NF-κB), mitogen-activated protein kinases (MAPKs), and nuclear receptors [5,6,7,8,9].

Synergistic interactions among phytochemicals have also been reported, leading to enhanced suppression of inflammatory mediators. Combined treatment with silibinin and dibenzoylmethane, for instance, produces synergistic anti-inflammatory effects in LPS-stimulated RAW264.7 cells by modulating the NF-κB and HIF-1 pathways [10]. Co-administration of chicoric acid and luteolin likewise attenuates inflammatory responses by inactivating the PI3K–Akt pathway and impairing NF-κB nuclear translocation [11]. Although such findings highlight the potential of combinatorial phytochemical approaches, the molecular basis of these synergistic effects—particularly how compound combinations modulate intracellular signaling networks in macrophages—remains insufficiently understood. Clarifying these mechanisms is crucial for the rational development of dietary or pharmacological interventions targeting inflammation.

To address this gap, we focused on plant-derived compounds that activate transient receptor potential (TRP) channels and possess anti-inflammatory properties. TRP channels constitute a family of ion channels with critical roles in numerous physiological processes, including immune modulation. They are widely expressed across sensory neurons, epithelial cells, and immune cells such as monocytes and macrophages. Upon activation by diverse stimuli, including temperature shifts, mechanical stress, and chemical irritants, TRP channels regulate intracellular calcium levels and trigger signaling cascades that promote the production of pro-inflammatory cytokines and chemokines [12].

Several TRP subtypes—such as TRPV1, TRPV4, TRPA1, TRPC3, and TRPM2—contribute to key immune functions, including phagocytosis, cytokine production, and cell survival [13]. Activation of these channels can stimulate signaling pathways such as NF-κB, MAPKs, and JAK/STAT, resulting in increased expression of IL-1, IL-6, TNF-α, and IL-17. In contrast, TRPM8 and TRPC5 have been associated with anti-inflammatory outcomes, illustrating the context-dependent nature of TRP channel function in immune regulation.

Given their broad role in immune modulation, TRP channels have attracted considerable interest as therapeutic targets for inflammatory diseases. However, it remains unclear whether and how combinations of plant-derived TRP channel agonists interact to modulate macrophage inflammatory responses. Therefore, in the present study, we investigated the anti-inflammatory effects of selected phytochemicals—including the TRPV1 agonist capsaicin (CA) [14], the TRPA1 agonist β-eudesmol (EU) [15], and the TRPM8 agonists menthol (ME) and 1,8-cineole (CI) [16,17]—both individually and in combination, using LPS-stimulated RAW264.7 macrophages, with the aim of elucidating the contribution of TRP-dependent and TRP-independent signaling pathways to their potential synergistic actions.

## 2. Materials and Methods

### 2.1. RAW264.7 Macrophage Cell Culture and Chemical Treatment

RAW264.7 cells were routinely cultured in 100 mm tissue culture dishes in Dulbecco’s Modified Eagle’s Medium (DMEM) containing 30 mg/mL glutamine and supplemented with 10% (*v*/*v*) fetal bovine serum, penicillin (100 units/mL), and streptomycin (100 µg/mL). Cells were maintained at 37 °C in a humidified incubator with 5% CO_2_ and allowed to attach and stabilize for 24 h. Cells were then harvested after 24 h of culture and seeded into 12-well plates at a density of 5 × 10^5^ cells per well in 1 mL of complete culture medium, followed by incubation for an additional 24 h to allow cell adherence and stabilization.

After this incubation period, cells were either subjected to calcium imaging analysis (see below) or further treated for 24 h by adding dimethyl sulfoxide (DMSO) to the culture medium (final concentration, 0.1% (*v*/*v*)), together with *l*-menthol (ME), 1,8-cineole (CI), β-eudesmol (EU), and capsaicin (CA) (FUJIFILM Wako Pure Chemical Corporation, Osaka, Japan) at the indicated concentrations, either individually or in defined combinations. A solution containing 0.1% (*v*/*v*) DMSO alone was used as the control solution.

To assess the effect of lipopolysaccharide (LPS) stimulation, cells were incubated with or without 1 μg/mL LPS (Sigma-Aldrich, St. Louis, MO, USA) in culture medium for 1, 3, or 6 h. A series of inhibitors/antagonists were applied, including *N*-(3-Aminopropyl)-2-[(3-methylphenyl)methoxy]-*N*-(2-thienylmethyl)benzamide (AMTB) hydrochloride (7.5 µM; FUJIFILM Wako Pure Chemical Corporation), a TRPM8 channel inhibitor; A-967079 (20 µM; FUJIFILM Wako Pure Chemical Corporation), a TRPA1 channel inhibitor; and AMG9810 (10 µM; FUJIFILM Wako Pure Chemical Corporation), a TRPV1 channel inhibitor. These compounds were added 2 h prior to the application of phytochemicals. A no-additive control group treated with culture medium alone was included in all experiments.

### 2.2. Cell Viability Assay

RAW264.7 cells were preincubated for 24 h and then treated with various concentrations of phytochemicals (final concentrations: 1–500 µM) for an additional 24 h. The cells were subsequently stained with propidium iodide (1 mg/mL; FUJIFILM Wako Pure Chemical Corporation) and analyzed using a BD FACSLyric™ flow cytometer (Becton, Dickinson and Company, Franklin Lakes, NJ, USA), according to previous studies [7,8].

### 2.3. RNA Isolation, cDNA Synthesis, and Quantitative Polymerase Chain Reaction (qPCR)

Total RNA was isolated from RAW264.7 cells using TRIzol^®^ reagent (Thermo Fisher Scientific, Waltham, MA, USA) according to the manufacturer’s protocol. First-strand cDNA was synthesized from 0.5 µg of total RNA using the ReverTra Ace qPCR RT Master Mix with gDNA Remover (Toyobo, Osaka, Japan). The RNA samples were first incubated at 37 °C for 5 min to eliminate genomic DNA, followed by reverse transcription at 37 °C for 15 min. qPCR was performed using a CFX Connect Real-Time PCR Detection System (Bio-Rad, Hercules, CA, USA) with THUNDERBIRD SYBR qPCR Mix (Toyobo) and gene-specific primers (Supplementary Table S1). The PCR cycling conditions were as follows: initial polymerase activation at 95 °C for 60 s, followed by 40 cycles of denaturation at 95 °C for 15 s and annealing/extension at 60 °C for 30 s. A melting curve analysis was performed using the instrument’s default settings. Relative gene expression levels were calculated after normalization to the reference gene *Gapdh*, following the method described in our previous studies [8,9].

### 2.4. Calcium Imaging

Intracellular calcium levels were monitored using the fluorescent calcium indicator Fluo-4 AM (Thermo Fisher Scientific). Cells cultured in 12-well plates were incubated with 1 mL of DMEM (FUJIFILM Wako Pure Chemical Corporation) containing 1 µM Fluo-4 AM at 27 °C in 5% CO_2_ for 1 h. After incubation, the medium was replaced with 1 mL of DMEM supplemented with 1 µM ionomycin (Sigma-Aldrich, St. Louis, MO, USA) as a positive control, or with phytochemicals (100 µM EU or CA, and 1 mM ME or CI). Fluorescence signals were recorded using an inverted fluorescence microscope (EVOS M7000 Imaging System, Thermo Fisher Scientific) with excitation at 482/25 nm and emission at 524/24 nm. Images were acquired at 1-s intervals.

### 2.5. Enzyme-Linked Immunosorbent Assay (ELISA)

TNF-α levels in the culture supernatants were quantified using a commercially available Mouse ELISA MAX™ Deluxe Set for Mouse TNF-α (BioLegend, San Diego, CA, USA) according to the manufacturer’s instructions. Briefly, RAW264.7 cells were treated as described above, and culture supernatants were collected and clarified by centrifugation to remove cell debris. ELISA plates were coated with capture antibody and incubated overnight at 4 °C, followed by blocking with the supplied blocking buffer. Samples and TNF-α standards were then added to the plates and incubated at room temperature. After washing, the detection antibody and avidin–horseradish peroxidase conjugate were sequentially applied. Color development was achieved using tetramethylbenzidine substrate and terminated by the addition of stop solution. Absorbance was measured at 450 nm, with background correction at 570 nm, using an iMark™ Microplate Reader (Bio-Rad, Hercules, CA, USA). TNF-α concentrations were calculated from a standard curve and expressed as pg mL^−1^.

### 2.6. Statistics and Reproducibility

We performed one-way analysis of variance (ANOVA) followed by Holm’s sequential Bonferroni post hoc test or Tukey’s post hoc honestly significant difference (HSD) using the multiple-sample comparison program available at http://astatsa.com/OneWay_Anova_with_TukeyHSD/ (accessed on 1 December 2025). When data did not meet the assumptions for parametric analysis, appropriate nonparametric tests were used. Sample sizes and the number of biological replicates for all experiments are provided in the legends of the corresponding figures.

## 3. Results

### 3.1. Effective Doses of Phytochemicals for Suppressing LPS-Induced TNF-α (Tnf) Gene Expression

**Figure 1:**
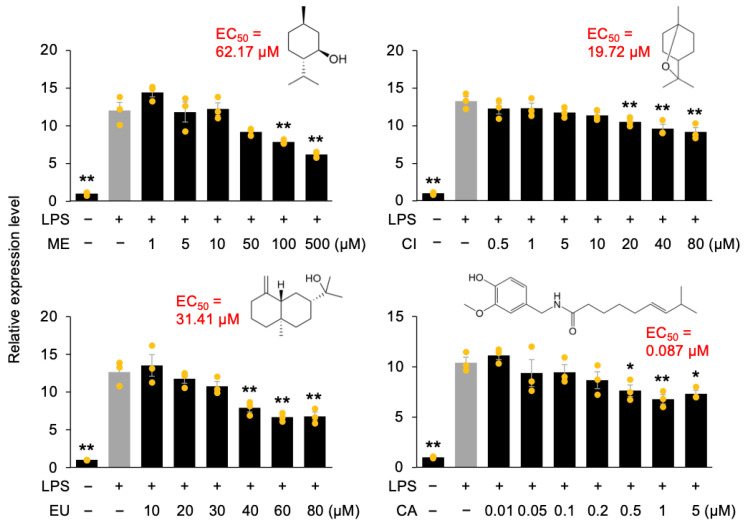
Anti-inflammatory effects of menthol (ME), 1,8-cineole (CI), β-eudesmol (EU), and capsaicin (CA) on *Tnf* expression in RAW264.7 cells. Cells were pretreated for 24 h with varying concentrations of each individual compound or with the control solution (0.1% (*v*/*v*) DMSO, −). Relative expression levels of *Tnf* were measured 1 h after stimulation with (+) or without (−) lipopolysaccharide (LPS). Data are presented as individual values along with means and standard errors (*n* = 3). Asterisks indicate statistically significant differences compared to the LPS-stimulated control group without compound treatment (gray columns), as determined by one-way ANOVA followed by Holm’s sequential Bonferroni post hoc test (**, *p* < 0.01; *, *p* < 0.05).

To evaluate anti-inflammatory activity, the expression levels of the tumor necrosis factor-alpha (TNF-α) gene (*Tnf*), which encodes a key proinflammatory cytokine, were measured in RAW264.7 cells pretreated with solutions containing ME, CI, EU, or CA, followed by stimulation with LPS for 1 h. The dose-dependent effects of these phytochemicals on LPS-induced *Tnf* expression were assessed to determine their half-maximal effective concentrations (EC_50_), which were calculated as 62.17 µM for ME, 19.72 µM for CI, and 31.41 µM for EU, markedly higher than that of CA (0.087 µM) (Figure 1). Concentrations up to 500 µM for ME and CI and up to 100 µM for EU and CA did not cause cytotoxicity in macrophage cells (Supplementary Figure S1). These findings suggest that the observed suppression of LPS-induced *Tnf* expression occurred at non-cytotoxic concentrations, indicating that the anti-inflammatory effects were not attributable to cell damage.

### 3.2. TRP-Dependent and TRP-Independent Regulation of Tnf Expression by Phytochemicals

**Figure 2:**
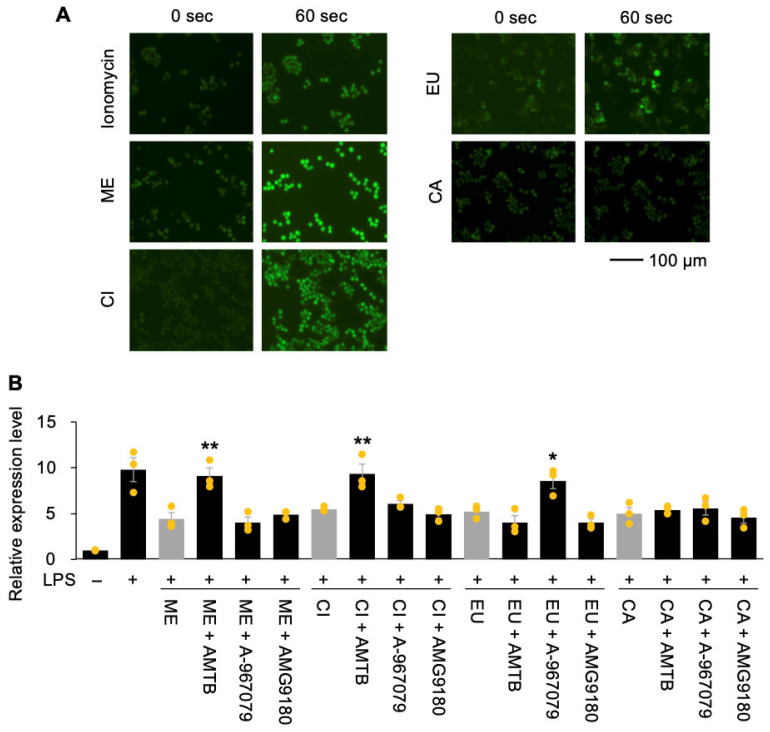
TRP-dependent and TRP-independent signaling in RAW264.7 cells in response to phytochemicals (menthol (ME), 1,8-cineole (CI), β-eudesmol (EU), and capsaicin (CA)). (**A**) Intracellular calcium influx in cells stimulated with ionomycin as a positive control or with the indicated phytochemicals. (**B**) Cells were treated with or without specific inhibitors/antagonists—AMTB hydrochloride (TRPM8 inhibitor), A-967079 (TRPA1 inhibitor), and AMG9810 (TRPV1 inhibitor)—and subsequently treated with phytochemicals. Relative expression levels of *Tnf* were measured 1 h after stimulation with (+) or without (−) lipopolysaccharide (LPS). Data are presented as individual points with means and standard errors (*n* = 3). Asterisks indicate statistically significant differences compared with the corresponding control group without inhibitor/antagonist treatment for each phytochemical (gray columns), as determined by one-way ANOVA followed by Holm’s sequential Bonferroni post hoc test (**, *p* < 0.01; *, *p* < 0.05).

As previously reported, the phytochemicals examined in this study have been identified as TRP channel agonists [14,15,16,17]. To determine whether the effects of phytochemicals on LPS-induced *Tnf* expression in RAW264.7 macrophages is mediated through TRP activation, we first monitored intracellular Ca^2+^ influx upon treatment with the phytochemicals. Treatment with the Ca^2+^ ionophore ionomycin produced a peak in intracellular Ca^2+^ influx at 60 s (Figure 2A). Similarly, treatment with high concentrations of ME, CL, or EU (100 µM or 1 mM) induced intracellular Ca^2+^ influx, whereas CA did not. Lower concentrations of the phytochemicals did not produce detectable fluorescence signals, likely due to the sensitivity limits of this system. These results suggest that ME, CL, and EU have the potential to activate TRP-mediated intracellular signaling pathways in RAW264.7 cells.

Next, we examined whether the inhibitory effects of these phytochemicals on LPS-induced *Tnf* expression depend on specific TRP channels in RAW264.7 cells. When cells were treated with AMTB hydrochloride, a TRPM8 inhibitor, the inhibitory effects of ME and CI were completely abolished, whereas inhibitors of other TRP channels did not affect their activity (Figure 2B). Similarly, treatment with A-967079, a TRPA1 inhibitor, abolished the inhibitory effect of EU. In contrast, none of the TRP inhibitors, including AMG9810 (a TRPV1 inhibitor), abolished the inhibitory effect of CA. Taken together, these results indicate that ME and CI regulate *Tnf* expression in a TRPM8-dependent manner, and EU acts through TRPA1, whereas CA modulates *Tnf* expression independently of any of the tested TRP channels, including TRPV1.

### 3.3. Synergistic Anti-Inflammatory Effects of ME or CI in Combination with CA

**Figure 3:**
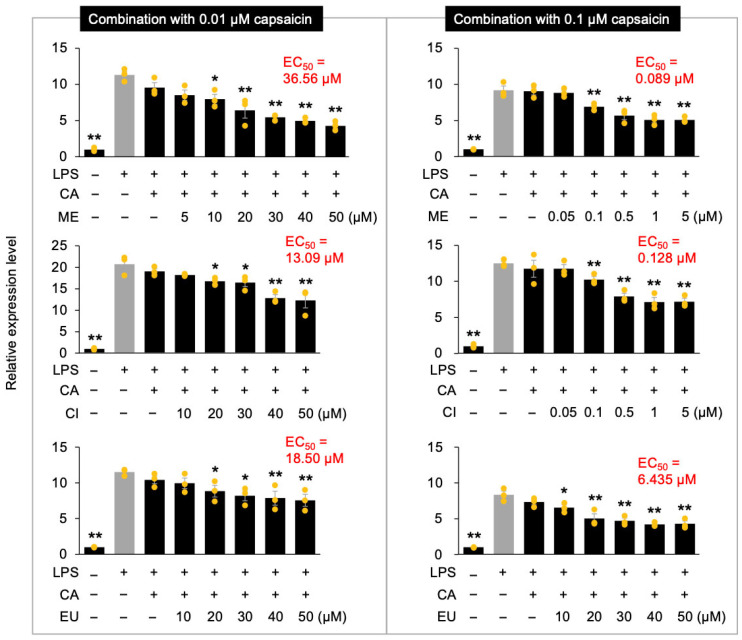
Anti-inflammatory effects of capsaicin (CA) in combination with menthol (ME), 1,8-cineole (CI), or β-eudesmol (EU) on *Tnf* expression in RAW264.7 cells. Cells were pretreated for 24 h with either 0.01 µM or 0.1 µM CA (+) or without CA (−), in combination with or without (−) varying concentrations of each individual compound. Relative expression levels of *Tnf* were measured 1 h after stimulation with (+) or without (−) lipopolysaccharide (LPS). Data are presented as individual values along with means and standard errors (*n* = 3). Asterisks indicate statistically significant differences compared to the LPS-stimulated control group without compound treatment (gray columns), as determined by one-way ANOVA followed by Holm’s sequential Bonferroni post hoc test (**, *p* < 0.01; *, *p* < 0.05).

We next investigated the potential synergistic anti-inflammatory effects arising from the combination of CA, a compound exhibiting a remarkably low EC_50_, with other phytochemicals. RAW264.7 cells were treated with mixtures containing either 0.1 µM CA (approximately its EC_50_ concentration) or a tenfold lower dose (0.01 µM), together with varying concentrations of ME, CI, or EU. The concentrations of the co-administered phytochemicals were selected based on their individual dose–response effects and were confirmed to be non-cytotoxic under the experimental conditions used (Supplementary Figure S2). The inhibitory effects on LPS-induced *Tnf* gene expression were subsequently assessed. Notably, neither 0.1 µM nor 0.01 µM CA alone produced a statistically significant suppression of *Tnf* expression in LPS-stimulated RAW264.7 cells (Figure 1). However, co-administration of 0.01 µM CA with the test phytochemicals resulted in EC_50_ values of 36.56 µM, 13.09 µM, and 18.50 µM for ME, CI, and EU, respectively. These correspond to approximately 1.7-, 1.5-, and 1.7-fold reductions compared with the EC_50_ values observed for each compound when applied individually (Figure 3).

**Figure 4:**
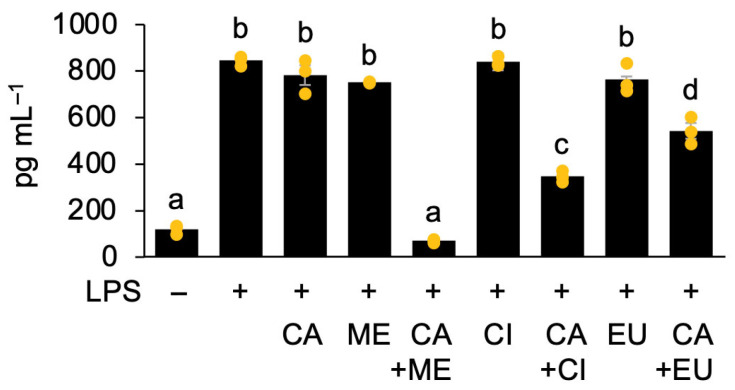
Synergistic effects of capsaicin (CA) with menthol (ME), 1,8-cineole (CI), or β-eudesmol (EU) on TNF-α protein levels in RAW264.7 cells. Cells were pretreated for 24 h with 0.1 µM CA in combination with 5 µM ME, 5 µM CI, or 30 µM EU. TNF-α levels were determined after stimulation with (+) or without (−) LPS for 3 h. Data are presented as individual values along with means and standard errors (*n* = 3). Means indicated by different lowercase letters are significantly different, as determined by one-way ANOVA followed by Tukey’s HSD post hoc test (*p* < 0.05).

More strikingly, upon co-administration with 0.1 µM CA, the EC_50_ of EU was reduced by 4.9-fold. The effects were even more pronounced for ME and CI, whose EC_50_ values decreased by 699-fold and 154-fold, respectively (Figure 3). These findings provide compelling evidence that ME and CI exhibit potent synergistic anti-inflammatory effects when co-administered with CA at specific concentrations (i.e., 0.1 µM). These results were consistent with the protein levels of TNF-α in LPS-stimulated cells following co-treatment with CA and ME, CI, or EU (Figure 4). Among these combinations, co-treatment with CA and ME was the most effective.

**Figure 5:**
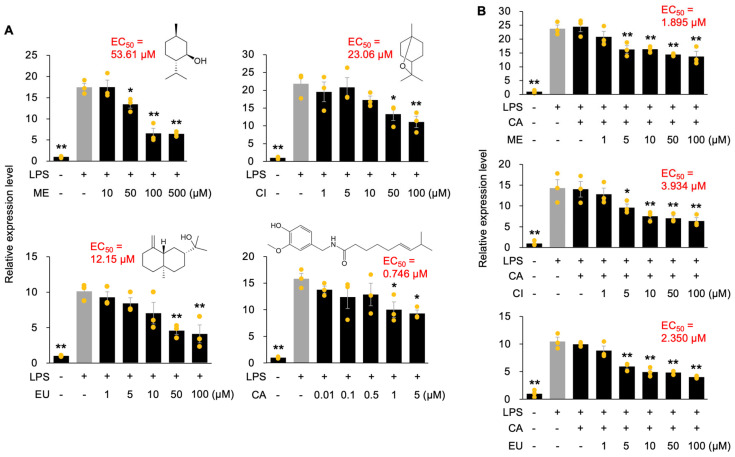
Anti-inflammatory effects of menthol (ME), 1,8-cineole (CI), β-eudesmol (EU), and capsaicin (CA) as well as CA in combination with ME, CI or EU on *Il6* (Interleukin-6 gene) expression in RAW264.7 cells. Cells were pretreated for 24 h with varying concentrations of each individual compound alone (without additional compounds, −) (**A**) or with varying concentrations of each individual compound co-administered with 0.1 µM CA (with additional compounds, +; without additional compounds, −) (**B**). Relative expression levels of *Il6* were measured 6 h after stimulation with (+) or without (−) lipopolysaccharide (LPS). Data are presented as individual values along with means and standard errors (*n* = 3). Asterisks indicate statistically significant differences compared to the LPS-stimulated control group without compound treatment (gray columns), as determined by one-way ANOVA followed by Holm’s sequential Bonferroni post hoc test (**, *p* < 0.01; *, *p* < 0.05).

Similar synergistic effects were also observed for another pro-inflammatory gene, *Il6*. Upon co-administration with 0.1 µM CA, the EC_50_ values of ME, CI, and EU were reduced by 28.3-, 5.9-, and 5.2-fold, respectively (Figure 5). As the magnitude of synergy was less pronounced than that observed for *Tnf* expression, these results suggest that the synergistic effects of CA are gene-dependent.

## 4. Discussion

In this study, we evaluated the anti-inflammatory activities of the phytochemicals ME, CI, EU, and CA by assessing their ability to suppress LPS-induced *Tnf* expression in RAW264.7 cells. Among the individual compounds, CA exhibited the strongest inhibitory effect, with the lowest EC_50_ value (0.087 µM). However, because RAW264.7 cells reportedly express little to no TRPV1 [18,19], CA is likely to modulate pro-inflammatory gene expression through TRPV1-independent pathways. Indeed, in our experiments, CA failed to induce calcium influx (Figure 2A), and inhibition of TRPV1 with AMG9810 did not alter the suppressive effect of CA on *Tnf* expression (Figure 2B). One potential TRPV1-independent mechanism is suggested by a recent study showing that CA ameliorates inflammation and the Warburg effect by directly targeting PKM2–LDHA and COX-2 in LPS-stimulated RAW264.7 cells [20]. Such alternative regulatory pathways may render cells more responsive to additional inputs from TRP-activating phytochemicals and, conversely, may themselves be influenced by these phytochemicals, thereby promoting the strong synergistic interactions observed in this study.

Co-treatment of CA with other phytochemicals revealed pronounced synergistic effects, particularly in LPS-stimulated RAW264.7 cells treated with ME or CI (Figure 3). During co-administration with 0.1 µM CA, EU exhibited an approximately fivefold reduction in the EC_50_ value for *Tnf* expression, whereas ME and CI showed striking reductions of approximately 700-fold and 150-fold, respectively. Notably, studies that quantitatively evaluate synergistic anti-inflammatory effects based on EC_50_ or IC_50_ values are limited, and the magnitude of synergy reported in such studies is generally modest. For example, a previous report on the combination of luteolin and chicoric acid demonstrated synergistic effects, with less than a one-order-of-magnitude reduction in IC_50_ (half maximal inhibitory concentration) values [11]. In this context, the approximately 700-fold reduction in EC_50_ observed for the CA + ME combination represents an exceptionally strong synergistic interaction that, to our knowledge, has rarely been documented in macrophage-based anti-inflammatory assays.

In contrast, for *Il6* expression, synergistic effects were observed but were less pronounced. ME and CI showed 28-fold and 6-fold decreases in EC_50_ values, respectively, upon co-administration with CA, and the CA + CI combination appeared to exhibit an effect closer to additive rather than synergistic (Figure 5). Taken together with the pronounced reductions in EC_50_ values for *Tnf* expression observed upon co-administration of CA with ME or CI, these findings suggest that TRP-independent PKM2–LDHA targeting by CA, combined with TRP-dependent signaling triggered by ME or CI, may differentially modulate cellular responsiveness in a gene-dependent manner, potentially through alterations in membrane permeability or intracellular signaling dynamics. This discrepancy is likely attributable to differences in promoter architecture and transcription factor requirements for each gene [21,22]. However, the molecular basis of this crosstalk remains unclear. Identifying which kinases or transcription factors—such as CaMK, AMPK, HIF-1α, NF-κB, or ROS-responsive pathways—mediate communication between TRP activation and PKM2/LDHA modulation will be essential for elucidating the full mechanistic framework.

Importantly, circulating concentrations of dietary phytochemicals rarely exceed the nanomolar range; therefore, the marked reduction in effective doses observed in this study may help reconcile the discrepancy between in vitro and in vivo efficacy. This notion is consistent with evidence that the health-promoting effects of polyphenol-rich diets arise from cumulative and synergistic interactions among multiple bioactive components [23,24]. Nonetheless, translating in vitro synergy into in vivo relevance will require rigorous evaluation using animal models and pharmacokinetic analyses. The substantial gap between the micromolar concentrations typically needed to elicit effects in vitro and the nanomolar bioavailability achieved through dietary intake remains a major challenge [25], underscoring the need to define physiologically achievable effective doses in vivo.

Furthermore, synergistic anti-inflammatory interactions among phytochemicals can produce robust responses at markedly lower concentrations than those required for each compound alone [25,26]. However, not all combinations enhance efficacy; mixtures may yield additive, synergistic, or even antagonistic outcomes depending on their molecular properties [23]. In this study, the CA + ME and CA + CI combinations clearly exhibited synergistic behavior, generating stronger anti-inflammatory effects than either compound alone.

## 5. Conclusions

In this study, we systematically examined the anti-inflammatory effects of selected plant-derived phytochemicals in LPS-stimulated RAW264.7 macrophages, with a particular focus on their combinatorial interactions. We demonstrated that specific phytochemical combinations produce pronounced synergistic anti-inflammatory effects, most notably a ~700-fold reduction in the EC_50_ value for *Tnf* expression upon co-treatment of CA with ME. This marked enhancement of efficacy was also observed for other combinations involving CA and occurred without detectable cytotoxicity. In addition to substantially increasing potency, phytochemical combinations therefore offer the advantage of achieving strong anti-inflammatory effects at lower effective doses of individual compounds, which may reduce the risk of adverse effects associated with high-dose single-compound treatments. Mechanistic analyses further suggest that this synergy arises from the integration of TRP-dependent signaling pathways activated by ME, CI, or EU with a TRP-independent pathway engaged by CA. Together, these findings highlight a cooperative mode of phytochemical action that markedly amplifies anti-inflammatory responses in macrophages and underscores the potential advantages of phytochemical combinations over single compounds for the control of inflammation.

Nevertheless, this study has several limitations. The experiments were conducted using an in vitro macrophage model, and the physiological relevance of the observed synergistic effects remains to be established in vivo. In addition, our analyses were limited to transcriptional changes in *Tnf* and *Il6*, without evaluation of protein-level responses for inflammatory mediators other than TNF-α or broader inflammatory markers. Moreover, although Ca^2+^ imaging and pharmacological inhibition provided insights into pathway involvement, the precise molecular intermediates connecting TRP-dependent and TRP-independent signaling pathways remain to be elucidated.

## Data Availability

The original contributions presented in this study are included in the article and Supplementary Materials. Further inquiries can be directed to the corresponding author.

## Supplementary Materials

The following supporting information can be downloaded at: https://www.mdpi.com/article/10.3390/nu18030376/s1, Figure S1: Viability of RAW264.7 cells following exposure to various concentrations of menthol (ME), 1,8-cineole (CI), β-eudesmol (EU), and capsaicin (CA); Figure S2: Viability of RAW264.7 cells following exposure to capsaicin (CA) in combination with menthol (ME), 1,8-cineole (CI), and β-eudesmol (EU); Table S1: Primers used for qPCR.

## Abbreviations

The following abbreviations are used in this manuscript:

ANOVA	Analysis of variance
CI	1,8-Cineole
CA	Capsaicin
EC_50_	half-maximal effective concentrations
ELISA	Enzyme-linked immunosorbent assay
EU	β-Eudesmol
*Il6*	Interleukin-6
ME	Menthol
LPS	Lipopolysaccharide
QPCR	Quantitative polymerase chain reaction
*Tnf*	Tumor necrosis factor-alpha
TRP	Transient receptor potential
