# Supplementary material for: Functional Phytochemicals Cooperatively Suppress Inflammation in RAW264.7 Cells
Source: Nutrients. 2026 Jan 23;18(3):376. doi: 10.3390/nu18030376 (PMC12899793; doi:10.3390/nu18030376)
Supplement: Supplementary file 1 [file nutrients-18-00376-s001.zip › Supplementalry Figures.pdf]

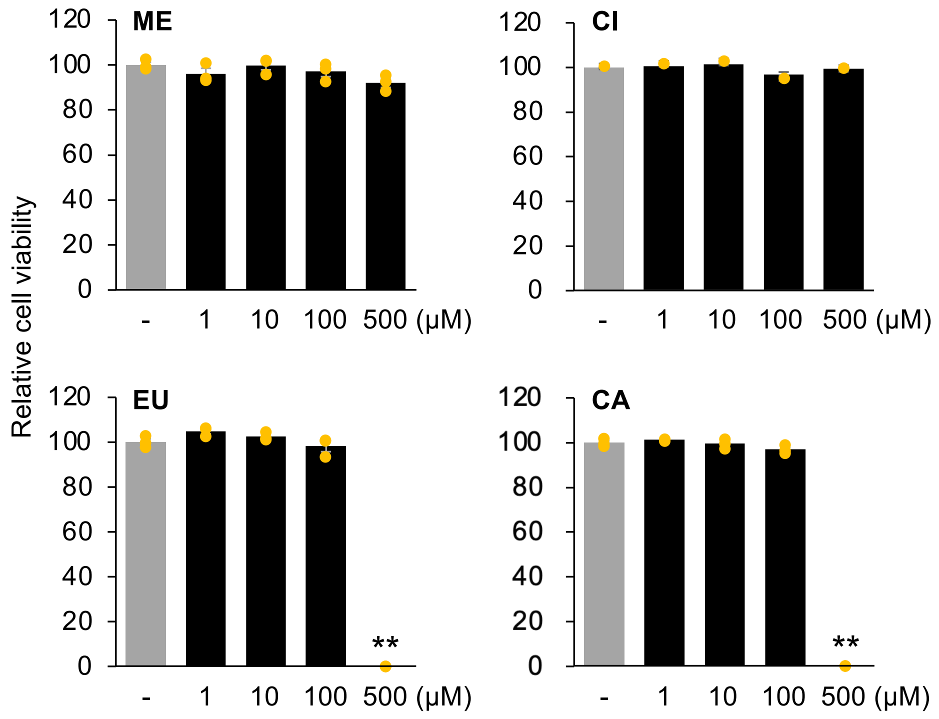

Supplementary Figure S1. Viability of RAW264.7 cells following exposure to various concentrations of menthol (ME), 1,8-cineole (CI), β-eudesmol (EU), and capsaicin (CA). Cell viability was assessed after 24 h of exposure to different concentrations of each individual compound, with untreated cells (−) serving as the control. Individual data points are shown along with mean ± standard error (n = 3). Asterisks indicate statistically significant differences compared to the untreated control (gray bars), as determined by one-way ANOVA followed by Holm's sequential Bonferroni post hoc test (**, $P < 0.01$).

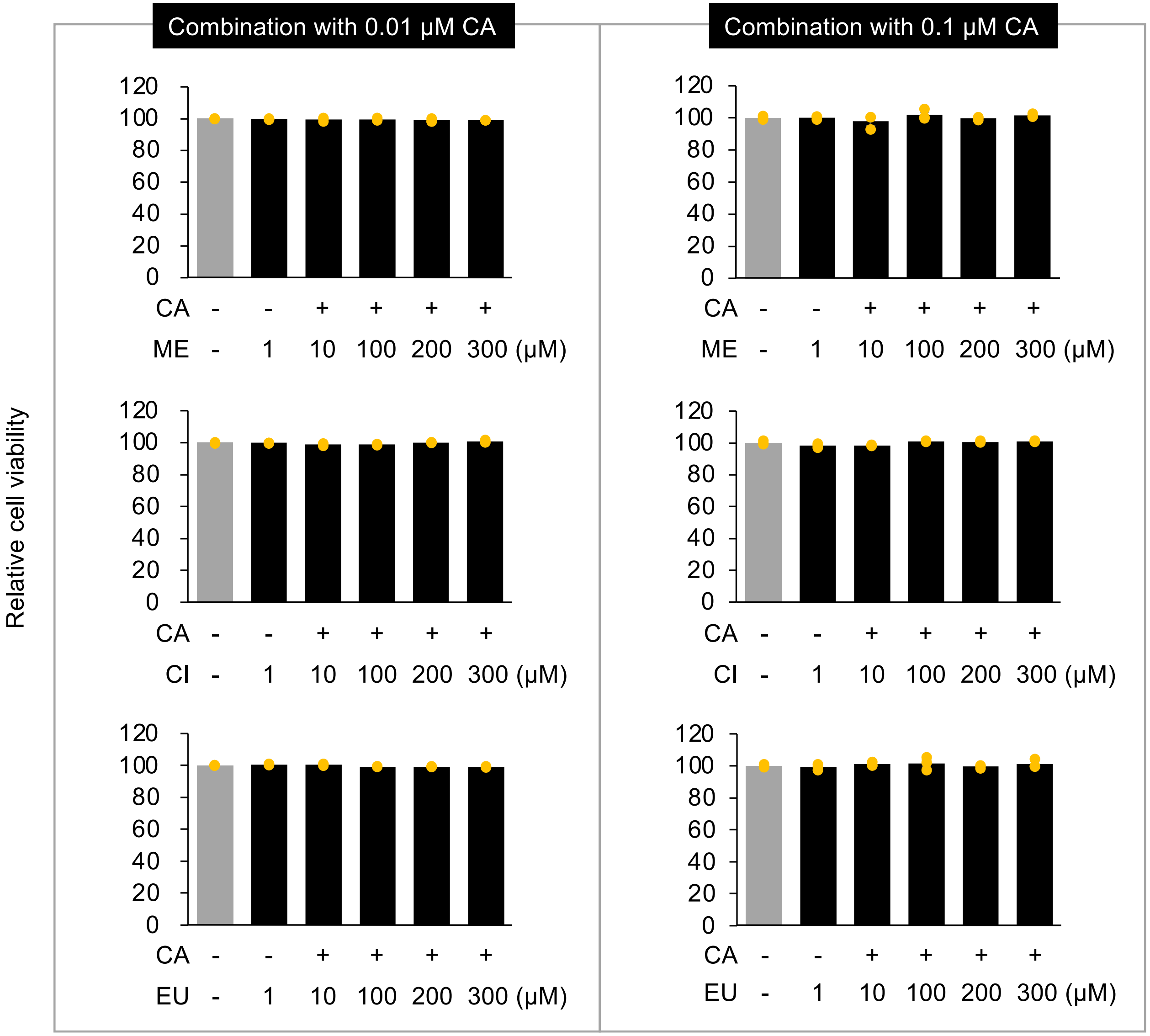

Supplementary Figure S2. Viability of RAW264.7 cells following exposure to capsaicin (CA) in combination with menthol (ME), 1,8-cineole (CI), and β-eudesmol (EU). Cell viability was assessed after a 24-hour exposure to either 0.01 μM or 0.1 μM CA (+) or no CA (−), in combination with varying concentrations of each individual compound or in the absence of the compounds (−). Individual data points are shown along with mean ± standard error (n = 3). Data are not significantly different from the untreated control (gray columns), as determined by one-way ANOVA followed by Holm's sequential Bonferroni post hoc test ($P > 0.05$).
